# Systemic Treatment Versus No Systemic Treatment After Biliary Drainage in Patients with Icteric Hepatocellular Carcinoma: A Retrospective Cohort Study

**DOI:** 10.3390/cancers18193138

**Published:** 2026-09-28

**Authors:** Chung-Wei Liu, Chi-Huan Wu, Yung-Kuan Tsou, Mu-Hsien Lee, Kai-Feng Sung, Cheng-Hui Lin

**Affiliations:** 1 Department of Gastroenterology and Hepatology, Linkou Chang Gung Memorial Hospital, Taoyuan 333, Taiwan; cwliu0216@gmail.com (C.-W.L.);; 2 College of Medicine, Chang Gung University, Taoyuan 333, Taiwan

**Keywords:** icteric hepatocellular carcinoma, obstructive jaundice, endoscopic retrograde cholangiopancreatography, systemic treatment

## Abstract

**Background/Objectives**: Icteric hepatocellular carcinoma (HCC) is characterized by obstructive jaundice, impaired liver function, and limited treatment options. Biliary drainage may relieve jaundice and permit subsequent anticancer therapy, but the clinical benefit of systemic treatment remains uncertain in this setting. **Methods:** This retrospective, single-center cohort study included 78 patients with icteric HCC who underwent biliary drainage via endoscopic retrograde cholangiopancreatography (ERCP) or percutaneous transhepatic cholangial drainage (PTCD). Thirty patients received systemic treatment during the treatment course surrounding biliary drainage (five initiated treatment before drainage and 25 on or after drainage), whereas 48 did not receive systemic treatment. Overall survival (OS) and progression-free survival (PFS) were evaluated using Kaplan–Meier analysis and Cox regression. Propensity score matching (PSM), time-dependent Cox regression, and a 42-day landmark analysis were performed as sensitivity analyses. **Results:** Median OS was longer with systemic treatment than without systemic treatment (14.6 vs. 6.7 months; *p* = 0.025), as was PFS (7.5 vs. 3.5 months; *p* = 0.005). After 1:1 PSM, systemic therapy remained associated with longer PFS (7.6 vs. 4.6 months; *p* = 0.044), whereas the OS difference was not statistically significant. In time-dependent analyses, systemic treatment was not significantly associated with OS (HR, 0.70; 95% CI, 0.40–1.22; *p* = 0.207) or PFS (HR, 0.65; 95% CI, 0.38–1.13; *p* = 0.128). **Conclusions:** Systemic treatment was associated with favorable outcomes in selected patients in the primary analyses, but these associations were attenuated after accounting for treatment timing. The findings are exploratory and do not establish a causal treatment effect.

## Simple Summary

Icteric hepatocellular carcinoma is rare and difficult to treat because biliary obstruction, impaired hepatic reserve, and advanced tumor burden frequently coexist. In this retrospective study of 78 patients undergoing biliary drainage, systemic treatment was associated with longer overall and progression-free survival in the primary analyses. After propensity score matching, the association with progression-free survival persisted, whereas the overall survival difference was not statistically significant. However, time-dependent Cox and 42-day landmark analyses accounting for treatment timing attenuated these associations and did not reach statistical significance. Radiotherapy was also associated with favorable outcomes, but treatment selection and timing biases preclude causal interpretation. These findings should therefore be considered exploratory and require validation in larger multicenter cohorts.

## 1. Introduction

Hepatocellular carcinoma (HCC) is the most common primary liver malignancy and remains a major global health burden [1,2]. Advanced-stage HCC commonly presents with vascular invasion, but rarely with obstructive jaundice. This uncommon presentation, caused by malignant bile duct invasion or obstruction, is referred to as icteric HCC and has been reported in 0.5–13% of HCC cases [3,4,5].

Icteric HCC is associated with a poor prognosis, with a reported median survival of 1.6–4.3 months among patients receiving palliative or best supportive care alone [3,6,7]. This unfavorable prognosis may be attributable to biliary obstruction, impaired hepatic reserve, and delays in initiating anticancer therapy. Current international guidelines provide no specific recommendations for the management of icteric HCC; therefore, treatment decisions are largely individualized [8,9,10,11].

Previous studies have suggested that biliary drainage procedures, including endoscopic retrograde cholangiopancreatography (ERCP) and percutaneous transhepatic cholangial drainage (PTCD), may relieve biliary obstruction and facilitate subsequent HCC treatment. In multicenter studies, successful biliary drainage has been associated with longer survival than unsuccessful drainage [4,5,12,13]. However, previous studies have focused primarily on drainage, whereas evidence supporting systemic treatment for icteric HCC remains limited and inconclusive.

Meanwhile, systemic treatment for advanced HCC has evolved rapidly, with tyrosine kinase inhibitors (TKIs) and immune checkpoint inhibitors (ICIs) demonstrating survival benefits in clinical trials [7,9,14,15,16,17]. However, patients with icteric HCC have generally been excluded from pivotal trials because of impaired liver function, as eligibility has traditionally been restricted to patients with Child–Pugh class A. Therefore, this study aimed to compare the clinical outcomes of patients with icteric HCC who received systemic treatment compared with those of patients managed without systemic treatment.

## 2. Materials and Methods

### 2.1. Study Design and Patient Selection

This retrospective, single-center cohort study was conducted at Linkou Chang Gung Memorial Hospital. We initially screened a database of 238 patients with HCC who underwent ERCP or PTCD between March 2001 and November 2024. The prespecified analytic period for the present study was January 2012 through November 2024; therefore, patients diagnosed with HCC before 2012 were excluded. Among the screened population, 133 consecutive patients with icteric HCC were identified, and 78 patients remained in the final analysis after application of the exclusion criteria (Figure 1). The exclusion criteria were as follows: (1) HCC diagnosed before 2012; (2) missing laboratory data; (3) combined hepatocellular–cholangiocarcinoma; (4) Barcelona Clinic Liver Cancer (BCLC) stage D [9]; and (5) previous surgical resection. All cases were reviewed at the institutional multidisciplinary HCC conference.

The study protocol was approved by the Institutional Review Board of Chang Gung Memorial Hospital (IRB No.: 202601230B0) and was conducted in accordance with the principles of the Declaration of Helsinki. Because of the retrospective nature of the study, the requirement for written informed consent was waived by the Institutional Review Board.

### 2.2. ERCP Protocol and Systemic Treatment Regimens

ERCP was performed under moderate sedation using a standard therapeutic duodenoscope (TJF-260 or JF-260; Olympus Optical Co., Tokyo, Japan). After selective biliary cannulation, endoscopic retrograde cholangiography was performed to identify the level and cause of biliary obstruction. Endoscopic sphincterotomy or endoscopic papillary balloon dilation was performed when indicated. Blood clots, tumor debris, and necrotic tissue within the bile duct were removed using a stone retrieval balloon to restore biliary drainage. In patients with residual biliary stricture after duct clearance, an 8.5 Fr plastic biliary stent (Flexima; Boston Scientific, Marlborough, MA, USA) was placed across the stricture to achieve adequate drainage.

Sorafenib was initiated orally at 400 mg once daily and could be escalated to 400 mg twice daily according to patient tolerability [14]. Lenvatinib was administered orally once daily at a body weight-based dose of 12 mg for patients weighing ≥60 kg and 8 mg for those weighing <60 kg [18]. Regorafenib was administered orally at 160 mg once daily for 3 weeks, followed by a 1-week rest period in each 4-week cycle [19]. Pembrolizumab was administered intravenously at 200 mg every 3 weeks [20]. Atezolizumab and bevacizumab were administered intravenously every 3 weeks at doses of 1200 mg and 5–15 mg/kg, respectively [21]. Nivolumab was administered intravenously at 3 mg/kg every 2 weeks [22]. Dose modifications were made at the discretion of the treating physicians, according to patient tolerability and the severity of adverse events. Treatment-related adverse events were graded according to the National Cancer Institute’s Common Terminology Criteria for Adverse Events 5.0 (CTCAE 5.0) [23].

Eligibility for systemic treatment was individualized, rather than determined by a single laboratory threshold. For patients in whom systemic treatment was initiated or continued after biliary drainage, treatment decisions were generally based on adequate biliary decompression, improvement or stabilization of cholestasis, sufficient performance status, and overall clinical condition to tolerate treatment. Some patients had already initiated systemic therapy before biliary drainage and continued treatment thereafter. Because systemic regimens and treatment lines varied over time, detailed individual regimens, treatment lines, timing relative to drainage, and post-drainage treatment durations are provided in Supplementary Table S3.

### 2.3. Definitions, Response Assessment, and Survival Outcomes

Icteric HCC was defined as HCC presenting with obstructive jaundice attributable to bile duct invasion or compression, as demonstrated by contrast-enhanced computed tomography (CT) or magnetic resonance imaging (MRI). Tumor response was assessed according to the modified Response Evaluation Criteria in Solid Tumors (mRECIST) [24]. Imaging follow-up was routinely scheduled approximately every 3 months. Additional imaging obtained outside the scheduled interval was also reviewed. Patients were categorized according to whether they received systemic therapy during the treatment course surrounding biliary drainage. The systemic treatment group included patients receiving TKI- or ICI-based treatment, either alone or in combination with locoregional treatment; 5 patients had initiated systemic therapy before drainage and 25 initiated treatment on or after drainage. The group without systemic treatment included patients receiving locoregional treatment, such as radiotherapy or transarterial chemoembolization (TACE), or best supportive care. For patients without available follow-up imaging, death from any cause was counted as a PFS event. Patients who remained alive without documented radiological progression were censored at the date of the last available disease assessment.

Overall survival (OS) was defined as the interval from the date of the index ERCP or PTCD procedure for icteric HCC to death from any cause or the last known follow-up date, defined as the most recent hospitalization or outpatient visit. Progression-free survival (PFS) was defined as the interval from the index ERCP or PTCD procedure to radiological tumor progression or death from any cause, whichever occurred first.

The Child–Pugh score was calculated using the lowest serum bilirubin level achieved after biliary drainage to reduce the influence of obstructive hyperbilirubinemia. Accordingly, this score represents a post-drainage hepatic functional assessment, rather than a true baseline measurement. Bilirubin normalization was defined as a serum total bilirubin level of ≤1.3 mg/dL after biliary drainage. Bilirubin normalization and other post-drainage bilirubin responses were therefore considered post-drainage clinical variables, rather than baseline characteristics. Acute cholangitis was diagnosed according to the Tokyo Guidelines 2018, based on evidence of systemic inflammation, cholestasis and characteristic imaging findings [25].

### 2.4. Statistical Analysis

Continuous variables are presented as medians with ranges and were compared using the Mann–Whitney U test. Categorical variables are presented as numbers and percentages and were compared using the chi-square test or Fisher’s exact test, as appropriate. Univariable and multivariable Cox proportional hazards regression models were used to analyze OS and PFS. Variables that were statistically significant in univariable analysis and considered clinically relevant were entered into the multivariable models. Kaplan–Meier analysis was used to estimate the survival probabilities, and the log-rank test was used to compare survival outcomes. All tests were two-sided, and *p*-value < 0.05 was considered statistically significant. Propensity scores were estimated using logistic regression including age, sex, portal vein thrombosis, extrahepatic spread, up-to-eleven criteria, and albumin–bilirubin (ALBI) grade [26]. Patients were matched 1:1 by nearest-neighbor matching without replacement using a caliper of 0.6 standard deviations of the propensity score distance. Covariate balance was evaluated using standardized mean differences (SMDs), with an absolute SMD < 0.10 used as the reference threshold for acceptable balance (Supplementary Table S5); balance before and after matching is additionally displayed in a Love plot (Supplementary Figure S1). Because residual imbalance in MELD score and hypertension remained after PSM, additional Cox models in the matched cohort adjusted for these variables. To address potential immortal time bias, systemic treatment was also modeled as a time-varying exposure; patients who initiated systemic therapy before or on the index drainage date were considered exposed from time zero, whereas patients initiating treatment after drainage contributed untreated person-time until systemic treatment initiation and treated person-time thereafter. A 42-day landmark analysis was additionally performed; patients who died before the landmark were excluded from the OS landmark analysis, and patients with progression or death before the landmark were excluded from the PFS landmark analysis. Statistical analyses were performed using R (version 4.6.1; R Foundation for Statistical Computing, Vienna, Austria) in RStudio (version 2025.09.1+401; Posit Software, PBC, Boston, MA, USA).

## 3. Results

### 3.1. Baseline Characteristics

A total of 78 patients with icteric hepatocellular carcinoma (HCC) who met the eligibility criteria were included. The median age of the overall cohort was 61 years, and 80.8% of the patients were male. Hepatitis B virus (HBV) and hepatitis C virus (HCV) infections were present in 60.2% and 21.8% of patients, respectively (Table 1).

Cirrhosis and esophageal varices were present in 78.2% and 33.3% of patients, respectively, and 48.7% had Child–Pugh class A. Because obstructive jaundice may overestimate the severity of hepatic dysfunction, we additionally evaluated bilirubin-excluded Child–Pugh. The between-group comparison of this modified score is presented in Table 1. Compared with the systemic treatment group, the group without systemic treatment had significantly higher MELD scores and more advanced ALBI grades.

The median maximum tumor size was 5.6 cm. Most patients had advanced-stage disease, with 66.7% classified as BCLC stage C. Portal vein thrombosis was present in 52.6% of patients, whereas macrovascular invasion and extrahepatic spread were observed in 57.7% and 30.8%, respectively. The median serum alpha-fetoprotein (AFP) level was 35.4 ng/mL.

Regarding biliary drainage, 65.4% of patients underwent endoscopic drainage by ERCP, whereas 34.6% underwent PTCD. The rates of drainage-related complications were generally comparable between the two groups. Drainage catheter or stent dislodgement occurred more frequently in the PTCD group, whereas the rates of other complications did not differ significantly between the two groups (Supplementary Table S1).

Combination treatment was administered more frequently in the systemic treatment group. Patients in the systemic treatment group also had more favorable bilirubin responses after biliary drainage, including higher rates of ≥20% reduction and normalization of serum bilirubin and lower post-drainage nadir bilirubin levels. However, the incidence of recurrent jaundice within 90 days did not differ significantly between the two groups. Baseline laboratory parameters, including white blood cell count, hemoglobin, platelet count, international normalized ratio (INR), creatinine, aspartate aminotransferase (AST), alanine aminotransferase (ALT), glutamyl transferase (γ-GT), alkaline phosphatase (ALK-P), albumin, and alpha-fetoprotein (AFP), were comparable between the groups.

In the systemic treatment group, the most frequently used regimens were sorafenib (10, 33.3%), lenvatinib (7, 23.3%), atezolizumab plus bevacizumab (5, 16.7%), and nivolumab (3, 10.0%); other regimens were used in one patient each. The median post-drainage systemic treatment duration was 78 days (IQR, 39–110 days; range, 18–1143 days). Detailed treatment sequences are provided in Supplementary Table S3. Among the 48 patients who did not receive systemic therapy, 18 received radiotherapy, nine received TACE, and three received both radiotherapy and TACE, whereas 24 received best supportive care only. The median interval from drainage to radiotherapy initiation was 30.5 days (IQR, 18.5–55.0 days; range, 0–87 days).

### 3.2. Efficacy Outcomes

The median OS for the entire cohort was 7.6 months (95% confidence interval [CI], 6.1–12.8). Median OS was longer in the systemic treatment group than in the group without systemic treatment (14.6 months [95% CI, 6.7–31.9] vs. 6.7 months [95% CI: 2.5–8.8]; *p* = 0.025) (Table 2 and Figure 2). The median PFS for the entire cohort was 4.1 months (95% CI, 3.3–7.6). Median PFS was also longer in the systemic treatment group than in the group without systemic treatment (7.5 months [95% CI, 4.1–27.4] vs. 3.5 months [95% CI, 1.9–7.3]; *p* = 0.005) (Table 2 and Figure 2). The median observed follow-up duration was 6.7 months (range 0.2–76.9 months).

Among the 30 patients receiving systemic treatment, five (16.7%) had initiated systemic therapy before biliary drainage and 25 (83.3%) initiated treatment on or after drainage. Among the latter, the median interval from biliary drainage to systemic treatment initiation was 42 days (IQR, 19–57 days; range, 0–88 days). In the time-dependent Cox analysis, systemic treatment was not significantly associated with OS (HR, 0.70; 95% CI, 0.40–1.22; *p* = 0.207) or PFS (HR, 0.65; 95% CI, 0.38–1.13; *p* = 0.128) (Supplementary Table S4). Similarly, in the 42-day landmark analysis, no significant association was observed for OS (HR, 1.07; 95% CI, 0.57–2.04; *p* = 0.826) or PFS (HR, 0.69; 95% CI, 0.34–1.39; *p* = 0.297). In conventional fixed-covariate models additionally adjusting for radiotherapy, systemic treatment was associated with lower hazards of death (adjusted HR, 0.55; 95% CI, 0.32–0.93; *p* = 0.027) and progression or death (adjusted HR, 0.48; 95% CI, 0.28–0.82; *p* = 0.007) (Supplementary Table S4); these estimates were interpreted cautiously because both treatments were predominantly administered after the index drainage procedure.

### 3.3. Prognostic Factors

In univariable Cox regression analysis (Table 3), systemic treatment was associated with a lower hazard of death (hazard ratio [HR], 0.550; 95% CI, 0.323–0.935; *p* = 0.027). Radiotherapy (HR, 0.365; 95% CI, 0.210–0.633; *p* = 0.001), bilirubin normalization after biliary drainage (HR, 0.466; 95% CI, 0.279–0.778; *p* = 0.004), and combination treatment (HR, 0.541; 95% CI, 0.308–0.951; *p* = 0.033) were also associated with lower mortality hazards. Because bilirubin normalization and treatment exposures occurred after the index drainage procedure, these associations were considered exploratory.

Conversely, Child–Pugh class B (HR, 2.274; 95% CI, 1.320–3.916; *p* = 0.003), Child–Pugh class C (HR, 2.973; 95% CI, 1.251–7.064; *p* = 0.014), and macrovascular invasion (HR, 2.161; 95% CI, 1.271–3.676; *p* = 0.004) were significantly associated with higher mortality hazards. Cirrhosis, BCLC stage, up-to-seven criteria, and biliary drainage modality were not significantly associated with OS.

In multivariable Cox regression analysis, systemic treatment remained associated with a lower mortality hazard (adjusted HR, 0.364; 95% CI, 0.181–0.736; *p* = 0.005), and radiotherapy was also associated with a lower mortality hazard (adjusted HR, 0.239; 95% CI, 0.105–0.541; *p* = 0.001). Child–Pugh class B was associated with a higher mortality hazard than Child–Pugh class A (adjusted HR, 2.126; 95% CI, 1.047–4.317; *p* = 0.037).

For Child-Pugh class C, the association observed in univariable analysis was attenuated and was no longer statistically significant after adjustment (adjusted HR 2.137; 95% CI, 0.747–6.115; *p* = 0.157). This estimate should be interpreted cautiously given the small number of patients with Child-Pugh class C (*n* = 8).

### 3.4. Propensity Score Matching

After 1:1 PSM, 25 patients were included in each group. Covariate balance improved after matching and is shown using SMDs and a Love plot (Supplementary Figure S1). However, residual imbalance remained for hypertension (24.0% vs. 60.0%; absolute SMD, 0.783) and MELD score (median, 9 vs. 13; absolute SMD 0.731) (Supplementary Table S2). These residual differences were considered evidence that PSM did not eliminate treatment selection bias.

In the propensity score-matched cohort, systemic treatment was associated with longer PFS than no systemic treatment (7.6 vs. 4.6 months; HR, 0.52; 95% CI, 0.27–0.98; *p* = 0.044). Median OS was numerically longer in the systemic treatment group, but the difference was not statistically significant (14.6 vs. 8.4 months; HR 0.71; 95% CI, 0.37–1.38; *p* = 0.315) (Table 2 and Figure 2). In an additional-adjusted analysis of the matched cohort accounting for residual imbalance in MELD score and hypertension, systemic treatment was not significantly associated with OS (adjusted HR, 0.68; 95% CI, 0.34–1.35; *p* = 0.265) but remained associated with a lower hazard of progression or death (adjusted HR, 0.50; 95% CI 0.25–0.98; *p* = 0.043).

## 4. Discussion

Icteric HCC represents a particularly challenging clinical condition in which biliary obstruction, impaired hepatic function and advanced tumor burden frequently coexist. In this setting, biliary drainage may provide symptomatic relief and may permit subsequent anticancer treatment [3,12]. In the primary analyses, patients who received systemic treatment had longer OS and PFS than patients managed without systemic treatment, and the PFS association persisted after PSM. However, the associations were attenuated and were no longer statistically significant in the time-dependent Cox and 42-day landmark analyses. Accordingly, the present findings should be interpreted as exploratory associations, rather than evidence of treatment efficacy.

Previous studies have primarily focused on the prognostic importance of successful biliary drainage. Woo et al. reported that complete resolution of jaundice and subsequent HCC treatment were associated with better survival in patients with advanced HCC with obstructive jaundice [4]. Matsumi et al. similarly demonstrated that successful endoscopic biliary drainage was associated with longer survival and a high technical success rate [5]. A recent multicenter study also identified successful biliary drainage as a favorable prognostic factor in patients with icteric HCC [12]. Our findings add real-world information regarding systemic treatment around biliary drainage, but the retrospective design and treatment selection process preclude determining whether biliary drainage itself enabled the observed survival differences.

Evidence regarding systemic treatment specifically for icteric HCC remains limited because patients with impaired hepatic function have generally been excluded from pivotal clinical trials. Tanaka et al. reported potential efficacy and acceptable safety of sorafenib in selected patients with unresectable HCC and bile duct invasion [27]. In the present cohort, systemic treatment included multiple TKI- and ICI-based regimens and different treatment lines, reflecting evolving real-world practice (Supplementary Table S3). This heterogeneity, together with the small number of patients receiving individual regimens, precludes regimen-specific efficacy conclusions. Recent real-world studies of Child–Pugh class B HCC have likewise shown heterogeneous outcomes with lenvatinib, atezolizumab plus bevacizumab, and nivolumab, emphasizing the importance of careful selection according to the degree of hepatic dysfunction [28,29,30].

Radiotherapy was associated with lower mortality in the conventional regression analyses. Suh et al. reported favorable outcomes after active HCC treatment, including TACE and radiotherapy, following effective biliary drainage [31], and proton beam therapy has demonstrated favorable local control in selected patients with unresectable HCC and bile duct invasion [32]. However, the use of radiotherapy in our retrospective cohort was subject to treatment selection bias and residual confounding. Therefore, the observed association between radiotherapy and survival should not be interpreted as evidence of a causal survival benefit or treatment synergy.

Hepatic recovery after biliary drainage appears clinically important when considering systemic treatment. Patients receiving systemic treatment generally had more favorable hepatic reserve and bilirubin response, whereas patients without systemic treatment had more ascites, higher MELD scores, and more advanced ALBI grades [33]. The bilirubin-excluded Child–Pugh score provides additional context in this population because obstructive cholestasis may inflate the conventional bilirubin component. However, the lowest post-drainage bilirubin used in the Child–Pugh calculation and bilirubin normalization are post-intervention variables and should not be interpreted as true baseline prognostic factors. Residual imbalance in MELD score and hypertension also remained after PSM, and additional regression adjustment did not eliminate the possibility of residual confounding.

This study has several limitations. First, its retrospective, single-center design and relatively small sample size may limit the generalizability of the findings. Second, treatment selection bias was unavoidable because patients selected for systemic treatment generally had better hepatic reserve and clinical stability. Although PSM and additional regression adjustment were performed, residual confounding remained. Third, the primary analysis was susceptible to immortal time bias because systemic and locoregional treatments were often initiated after the index drainage procedure. Time-dependent and landmark analyses attenuated the observed associations, underscoring the importance of cautious interpretation. Fourth, the no systemic treatment group was heterogeneous and included radiotherapy, TACE, and best supportive care; the sample size was insufficient. Fifth, systemic regimen, treatment lines, and standards of HCC care changed substantially between 2012 and 2024, creating potential treatment era confounding. Larger prospective, multicenter studies with standardized treatment and follow-up protocols are needed to validate these findings.

## 5. Conclusions

In this retrospective cohort, systemic treatment was associated with favorable OS and PFS in the primary analyses, whereas the OS association was not statistically significant after PSM and both OS and PFS associations were attenuated in analyses accounting for treatment timing. These findings should therefore be considered exploratory and hypothesis-generating, rather than evidence of causal treatment efficacy. Radiotherapy was also associated with outcomes in conventional analyses, but treatment selection and timing biases preclude causal inference. Larger prospective multicenter studies are needed to define the roles of systemic and multidisciplinary treatment strategies in patients with icteric HCC.

## Figures and Tables

**Figure 1:**
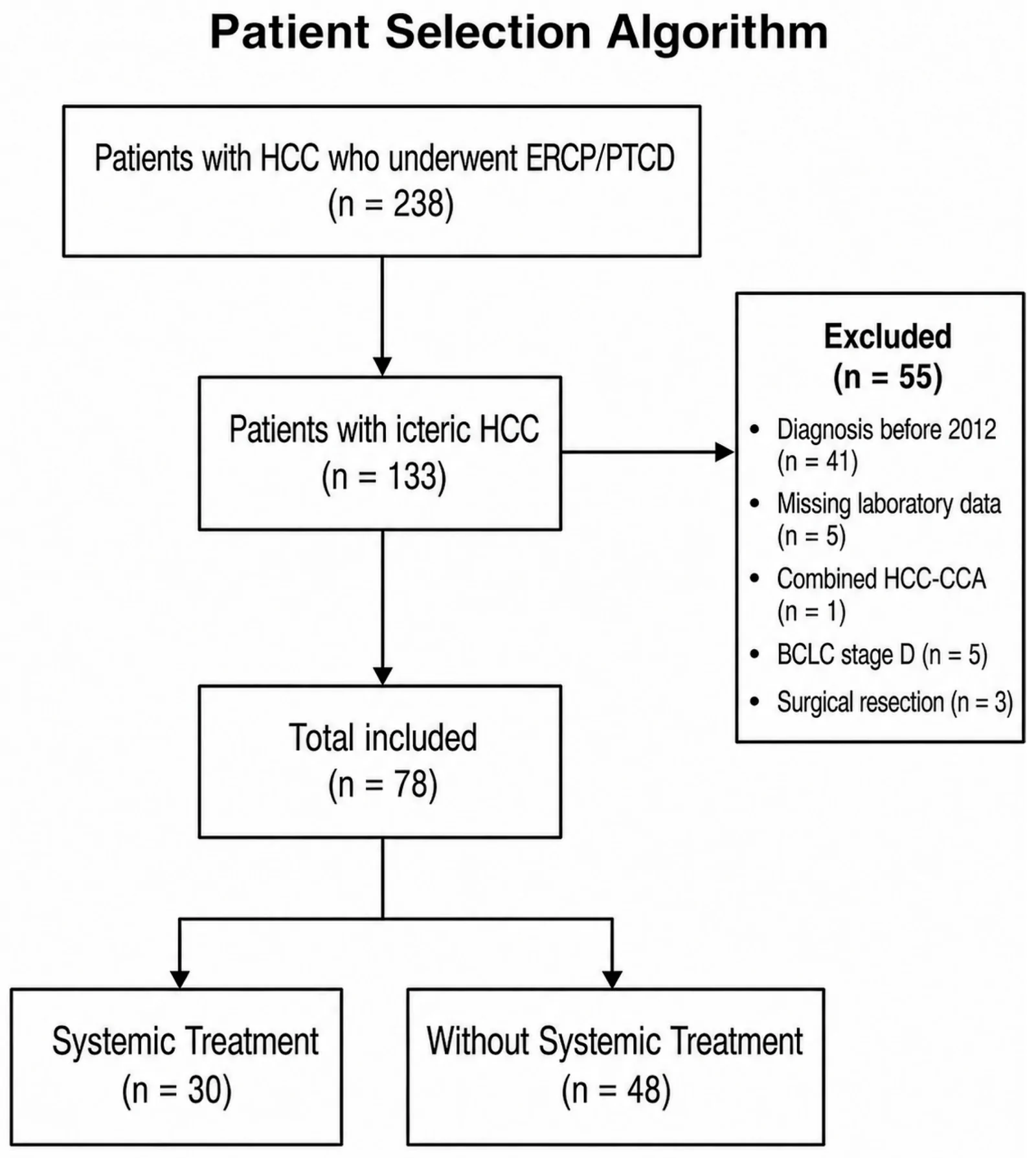
Patient selection algorithm. Abbreviations: HCC, hepatocellular carcinoma; ERCP, endoscopic retrograde cholangiopancreatography; PTCD, percutaneous transhepatic cholangial drainage; HCC-CCA, hepatocellular–cholangiocarcinoma; BCLC, Barcelona Clinic Liver Cancer.

**Figure 2:**
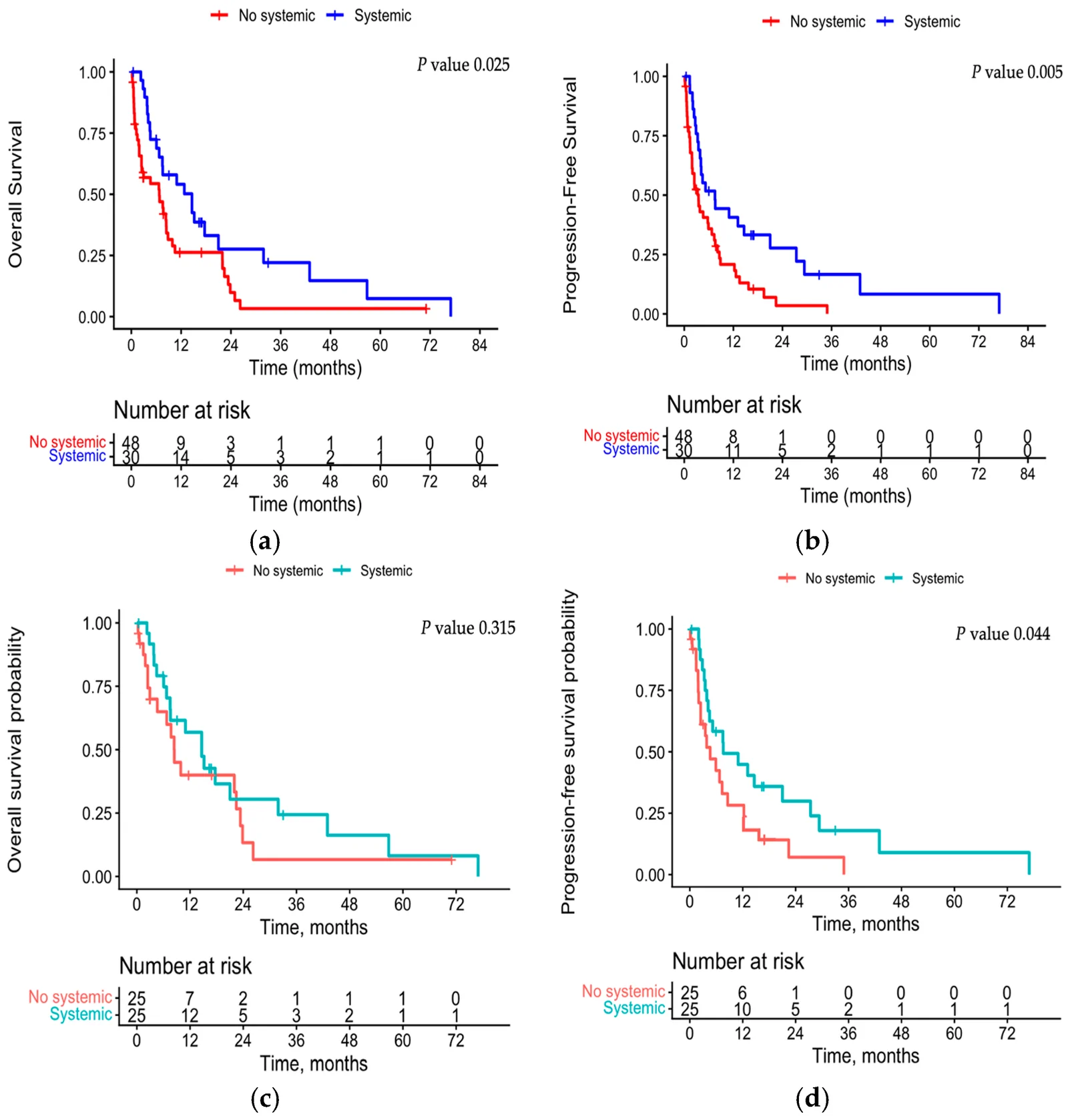
Clinical outcomes of systemic treatment versus no systemic treatment groups. (**a**) Overall survival; (**b**) progression-free survival; (**c**) overall survival after propensity score matching; (**d**) progression-free survival after propensity score matching.

**Table 1:** Baseline characteristics of patients receiving systemic treatment versus without systemic treatment.

Variables	All (*n* = 78)	Group		*p* Value
		Systemic Treatment (*n* = 30)	Without Systemic Treatment (*n* = 48)	
Male, *n* (%)	63 (80.8)	23 (76.7)	40 (83.3)	0.559
Age (years) *	61 (36–89)	59 (36–80)	62 (39–89)	0.223
HTN, *n* (%)	35 (44.9)	7 (23.3)	28 (58.3)	0.005
DM, *n* (%)	22 (28.2)	9 (30.0)	13 (27.1)	0.801
Alcoholism, *n* (%)	21 (26.9)	9 (30.0)	12 (25.0)	0.794
Fatty liver, *n* (%)	16 (20.5)	6 (20.0)	10 (20.8)	1.000
Viral hepatitis, *n* (%)	HBV 47 (60.2) HCV 17 (21.8) Both 2 (2.6)	HBV 19 (63.3) HCV 6 (20.0) Both 1 (3.3)	HBV 28 (58.3) HCV 11 (22.9) Both 1 (2.1)	0.899
Cirrhosis, *n* (%)	61 (78.2)	21 (70.0)	40 (83.3)	0.259
Ascites, *n* (%)	26 (33.3)	5 (16.7)	21 (43.8)	0.015
Child–Pugh score, *n* (%)	A 38 (48.7) B 32 (41.0) C 8 (10.3)	A 22 (73.3) B 7 (23.3) C1 (3.3)	A 16 (33.3) B 25 (52.1) C 7 (14.6)	0.001
Bilirubin-excluded Child–Pugh score *	5 (3–8)	5 (3–7)	5 (4–8)	0.386
EV, *n* (%)	26 (33.3)	9 (30.0)	17 (35.4)	0.805
AJCC stage, *n* (%)	I 12 (15.4) II 6 (7.7) III 34 (43.6) IV 26 (33.3)	I 2 (6.7) II 2 (6.7) III 14 (46.7) IV 12 (40.0)	I 10 (20.8) II 4 (8.3) III 20 (41.7) IV 14 (29.2)	0.085
BCLC stage, *n* (%)	0 3 (3.8) A 9 (11.5) B 14 (17.9) C 52 (66.7)	0 0 (0) A 3 (10.0) B 4 (13.3) C 23 (76.7)	0 3 (6.3) A 6 (12.5) B 10 (20.8) C 29 (60.4)	0.350
Maximum tumor size, (cm) *	5.6 (1.0–21.0)	6.1 (2.1–19.9)	5.2 (1.0–21.0)	0.163
Up-to-7, *n* (%)	56 (71.8)	24 (80.0)	32 (66.7)	0.301
Up-to-11, *n* (%)	53 (67.9)	23 (76.7)	30 (62.5)	0.221
PVT stage, *n* (%)	Nil 37 (47.4) I-II 5 (6.4) III-IV 36 (46.2)	Nil 13 (43.3) I-II 2 (6.7) III-IV 15 (50.0)	Nil 24 (50.0) I-II 3 (6.3) III-IV 21 (43.8)	0.846
MVI, *n* (%)	45 (57.7)	18 (60.0)	27 (56.3)	0.816
Extrahepatic Spread, *n* (%)	24 (30.8)	12 (40.0)	12 (25.0)	0.209
MELD score *	11 (6–37)	9 (6–18)	15 (7–37)	0.001
FIB-4 ≥ 3.25, *n* (%)	28 (35.9)	8 (26.7)	20 (41.7)	0.228
ALBI grade, *n* (%)	I 11 (14.1) II 44 (56.4) III 23 (29.5)	I 6 (20.0) II 21 (70.0) III 3 (10.0)	I 5 (10.4) II 23 (47.9) III 20 (41.7)	0.011
**Bile duct** **intervention**				
ERCP/PTCD, *n* (%)	51/27 (65.4/34.6)	20/10 (66.7/33.3)	31/17 (64.6/35.4)	1.000
Hemobilia, *n* (%)	17 (21.8)	6 (20.0)	11 (22.9)	1.000
Cholangitis, *n* (%)	54 (69.2)	21 (70.0)	33 (68.8)	1.000
**Post-drainage bilirubin assessment**				
Baseline, (mg/dL) *	12.2 (0.8–30.3)	13.6 (0.8–30.3)	11.5 (0.9–28.9)	0.642
Post-1 week intervention, (mg/dL) *	6.1 (0.5–28.4)	4.3 (0.5–16.2)	7.4 (0.7–28.4)	0.069
Post-intervention, nadir (mg/dL) *	1.6 (0.2–26.3)	0.9 (0.2–19.2)	3.0 (0.5–26.3)	0.001
Decrease ≥ 20%, *n* (%)	63 (80.8)	29 (96.7)	34 (70.8)	0.006
Normalization, *n* (%)	35 (44.9)	20 (66.7)	15 (31.3)	0.003
Recurrent jaundice within 90 days, *n* (%)	47 (60.3)	18 (60.0)	29 (60.4)	1.000
**Laboratory data**				
WBC (10^3^ cells/µL) *	7.0 (1.9–27.6)	7.2 (4.0–27.6)	6.8 (1.9–15.3)	0.642
Hb (g/dL) *	10.5 (6.1–16.7)	11.2 (6.1–16.7)	10.4 (6.9–15.8)	0.115
Platelet (103/µL) *	183 (32–830)	214 (92–677)	180 (32–830)	0.642
INR *	1.2 (1.0–1.9)	1.2 (1.0–1.5)	1.2 (1.1–1.9)	0.494
Creatinine (mg/dL) *	0.71 (0.26–3.80)	0.62 (0.26–1.44)	0.78 (0.36–3.80)	0.092
AST (U/L) *	71 (20–603)	64 (23–603)	84 (20–313)	0.163
ALT (U/L) *	63 (19–565)	55 (19–252)	80 (22–565)	0.063
γ -GT (U/L) *	225 (33–860)	243 (86–860)	222 (33–802)	0.530
ALK-P (U/L) *	221 (67–1305)	225 (67–672)	221 (76–1305)	1.000
Albumin (g/dL) *	3.3 (1.8–4.5)	3.4 (2.7–4.3)	3.3 (1.8–4.5)	0.352
AFP (ng/mL) *	35.4 (2.0–4,209,873.0)	84.1 (2.0–415,805.1)	34.2 (2.0–4,209,873.0)	0.642
**Treatment**				
RT, *n* (%)	34 (43.6)	16 (53.3)	18 (37.5)	0.241
TACE, *n* (%)	11 (14.1)	2 (6.7)	9 (18.8)	0.188
Combined treatment, *n* (%)	23 (29.5)	19 (63.3)	4 (8.3)	0.001

Abbreviations: HTN, hypertension; DM, diabetes mellitus; HBV, hepatitis B virus; HCV, hepatitis C virus; EV, esophageal varices; AJCC, American Joint Committee on Cancer; BCLC, Barcelona Clinic Liver Cancer; PVT, portal vein thrombosis; MVI, macrovascular invasion; MELD, Model for End-Stage Liver Disease; FIB-4, fibrosis-4 index; ALBI, albumin–bilirubin; ERCP, endoscopic retrograde cholangiopancreatography; PTCD, percutaneous transhepatic cholangial drainage; WBC, white blood cell count; Hb, hemoglobin; INR, international normalized ratio; AST, aspartate aminotransferase; ALT, alanine aminotransferase; γ-GT, gamma-glutamyl transferase; ALK-P, alkaline phosphatase; AFP, alpha-fetoprotein; RT, radiotherapy; TACE, transarterial chemoembolization. * Data are presented as median (range). The Child–Pugh score was calculated using the lowest serum bilirubin value achieved after biliary drainage and therefore represents a post-drainage hepatic functional assessment. Post-1 week bilirubin, post-intervention nadir bilirubin, bilirubin decrease, and bilirubin normalization are post-drainage variables, rather than true baseline characteristics. γ-GT had missing data (*n* = 17 of entire cohort).

**Table 2:** Survival outcomes of systemic treatment versus without systemic treatment.

**Survival** **Outcomes**	**All** (***n* = 78**)	**Systemic** **Treatment** (***n* = 30**)	**Without** **Systemic Treatment** (***n* = 48**)	***p* Value**
Overall survival (months, 95% CI)	7.6 (6.1–12.8)	14.6 (6.7–31.9)	6.7 (2.5–8.8)	0.025
Progression free survival (months, 95% CI)	4.1 (3.3–7.6)	7.5 (4.1–27.4)	3.5 (1.9–7.3)	0.005
**Propensity score matching**		**Systemic** **Treatment** (***n* = 25**)	**Without** **Systemic Treatment** (***n* = 25**)	***p* value**
Overall survival (months, 95% CI)		14.6 (7.5–56.8)	8.4 (4.6–23.8)	0.315
Progression free survival (months, 95% CI)		7.6 (4.5–29.4)	4.6 (2.5–12.6)	0.044

Abbreviations: CI, confidence interval.

**Table 3:** Cox regression analysis of predictors of overall survival in the entire cohort.

Variables		Crude HR (95% CI)	*p*	Adjusted HR (95% CI)	*p*
Treatment	Not systemic	Referent		Referent	
	Systemic	0.550 (0.323–0.935)	0.027	0.364 (0.181–0.736)	0.005
Cirrhosis	No	Referent			
	Yes	1.202 (0.654–2.211)	0.554		
Child–Pugh Score	A	Referent		Referent	
	B	2.274 (1.320–3.916)	0.003	2.126 (1.047–4.317)	0.037
	C	2.973 (1.251–7.064)	0.014	2.137 (0.747–6.115)	0.157
BCLC stage	0	Referent		Referent	
	A	0.557 (0.110–2.812)	0.850	0.521 (0.096–2.831)	0.450
	B	0.967 (0.211–4.432)	0.965	0.455 (0.082–2.528)	0.368
	C	1.599 (0.385–6.644)	0.518	1.508 (0.259–8.799)	0.648
Up-to-7	No	Referent		Referent	
	Yes	1.494 (0.832–2.681)	0.179	1.074 (0.522–2.212)	0.846
MVI	No	Referent		Referent	
	Yes	2.161 (1.271–3.676)	0.004	0.763 (0.254–2.292)	0.630
Intervention	ERCP	Referent			
	PTCD	0.766 (0.448–1.309)	0.329		
Bilirubin normalization	No	Referent		Referent	
	Yes	0.466 (0.279–0.778)	0.004	0.730 (0.354–1.506)	0.394
Combined treatment	No	Referent		Referent	
	Yes	0.541 (0.308–0.951)	0.033	1.376 (0.569–3.331)	0.479
RT	No	Referent		Referent	
	Yes	0.365 (0.210–0.633)	0.001	0.239 (0.105–0.541)	0.001

Abbreviations: HR, hazard ratio; CI, confidence interval; BCLC, Barcelona Clinic Liver Cancer; MVI, macrovascular invasion; ERCP, endoscopic retrograde cholangiopancreatography; PTCD, percutaneous transhepatic cholangial drainage; RT, radiotherapy.

## Data Availability

The data presented in this study are available from the corresponding author upon reasonable request.

## Supplementary Materials

The following supporting information can be downloaded at: https://www.mdpi.com/article/10.3390/cancers18193138/s1, Table S1: Post-interventional complications for patients receiving biliary drainage; Table S2: Baseline characteristics of patients receiving systemic treatment versus without systemic treatment after propensity score matching; Table S3: Individual systemic treatment regimens, treatment lines, timing relative to biliary drainage, post-drainage treatment duration, and subsequent systemic therapies; Table S4: Sensitivity analyses of the association between systemic treatment and survival outcomes; Table S5: Standardized mean differences before and after propensity-score matching; Figure S1: Love plot showing standardized mean differences before and after propensity score matching.

## Abbreviations

AFP	Alpha-fetoprotein
AJCC	American Joint Committee on Cancer
ALBI	Albumin–bilirubin
ALK-P	Alkaline phosphatase
ALT	Alanine aminotransferase
AST	Aspartate aminotransferase
BCLC	Barcelona Clinic Liver Cancer
CI	Confidence interval
CT	Computed tomography
CTCAE	Common Terminology Criteria for Adverse Events
DM	Diabetes mellitus
ERCP	Endoscopic retrograde cholangiopancreatography
EV	Esophageal varices
FIB-4	Fibrosis-4 index
γ-GT	Gamma-glutamyl transferase
Hb	Hemoglobin
HBV	Hepatitis B virus
HCC	Hepatocellular carcinoma
HCC-CCA	Combined hepatocellular carcinoma–cholangiocarcinoma
HCV	Hepatitis C virus
HR	Hazard ratio
HTN	Hypertension
ICI	Immune checkpoint inhibitor
INR	International normalized ratio
IRB	Institutional Review Board
MELD	Model for End-Stage Liver Disease
MRI	Magnetic resonance imaging
mRECIST	Modified Response Evaluation Criteria in Solid Tumors
MVI	Macrovascular invasion
OS	Overall survival
PFS	Progression-free survival
PSM	Propensity score matching
PTCD	Percutaneous transhepatic cholangial drainage
PVT	Portal vein thrombosis
RT	Radiotherapy
TACE	Transarterial chemoembolization
TKI	Tyrosine kinase inhibitor
WBC	White blood cell count
