# Supplementary material for: Systemic Treatment Versus No Systemic Treatment After Biliary Drainage in Patients with Icteric Hepatocellular Carcinoma: A Retrospective Cohort Study
Source: Cancers (Basel). 2026 Sep 28;18(19):3138. doi: 10.3390/cancers18193138 (PMC13664453; doi:10.3390/cancers18193138)
Supplement: Supplementary file 1 [file cancers-18-03138-s001.zip › cancers-4541908-supplementary.pdf]

Supplementary Table S1. Post Interventional Complications for Patients Receiving Biliary Drainage

Variables	All (N=78)	Group		*P* value
		ERBD (N=51)	PTCD (N=27)	
Fever, n (%)	15 (19.2)	12 (23.5)	3 (11.1)	0.237
Bleeding, n (%)	1 (1.3)	1 (2.0)	0	1.000
Infection, n (%)	5 (6.4)	5 (9.8)	0	0.157
Pancreatitis, n (%)	1 (1.3)	1 (2.0)	0	1.000
Bile duct injury, n (%)	2 (2.6)	0	2 (7.4)	0.117
Drainage tube/stent dislodge, n (%)	16 (20.5)	3 (5.9)	13 (48.1)	0.001

Abbreviations: ERCP, endoscopic retrograde cholangiopancreatography; PTCD, percutaneous transhepatic cholangial drainage;

Supplementary Table S2. Baseline characteristics of patients receiving systemic treatment versus without systemic treatment after propensity score matching

Variables	Systemic Group (N=25)	Without Systemic Group (N=25)	*P* value
Male, n (%)	21 (84.0)	21 (84.0)	1.000
Age (years)*	60 (36-80)	63 (39-84)	0.705
HTN, n (%)	6 (24.0)	15 (60.0)	0.021
DM, n (%)	7 (28.0)	7 (28.0)	1.000
Alcoholism, n (%)	9 (36.0)	6 (24.0)	0.538
Fatty liver, n (%)	6 (24.0)	7 (28.0)	1.000
Virus hepatitis, n (%)	HBV 15 (60.0)	HBV 12 (48.0)	0.721
	HCV 3 (12.0)	HCV 6 (24.0)	
	Both 1 (4.0)	Both 1 (4.0)	
Cirrhosis, n (%)	17 (68.0)	20 (80.0)	0.520
Ascites, n (%)	4 (16.0)	7 (28.0)	0.496
Child-Pugh score, n (%)	A 17 (68.0)	A 12 (48.0)	0.302
	B 7 (28.0)	B 10 (40.0)	
	C 1 (4.0)	C 3 (12.0)	
EV, n (%)	7 (28.0)	5 (20.0)	0.742
AJCC stage, n (%)	I 2 (8.0)	I 5 (20.0)	0.577
	II 2 (8.0)	II 2 (8.0)	
	III 13 (52.0)	III 13 (52.0)	
	IV 8 (32.0)	IV 5 (20.0)	

Variables	Systemic Group (N=25)	Without Systemic Group (N=25)	*P* value
BCLC stage, n (%)	0 0 (0.0)<br>A 3 (12.0)<br>B 4 (16.0)<br>C 18 (72.0)	0 2 (8.0)<br>A 2 (8.0)<br>B 9 (36.0)<br>C 12 (48.0)	0.150
Maximum tumor size (cm)*	5.7 (2.1-19.9)	6.5 (1.5-21)	0.734
Up-to-7, n (%)	19 (76.0)	19 (76.0)	1.000
Up-to-11, n (%)	18 (72.0)	18 (72.0)	1.000
PVT Nil/I-II/III-IV, n (%)	Nil 12 (48.0)<br>I-II 2 (8.0)<br>III-IV 11 (44.0)	Nil 13 (52.0)<br>I-II 2 (8.0)<br>III-IV 10 (40.0)	0.957
MVI, n (%)	14 (56.0)	12 (48.0)	0.778
Extrahepatic Spread, n (%)	7 (28.0)	4 (16.0)	0.496
MELD score*	9 (6-18)	13 (7-26)	0.036
FIB-4 ≥ 3.25, n (%)	6 (24.0)	6 (24.0)	1.000
ALBI stage I/II/III	I 5 (20.0)<br>II 17 (68.0)<br>III 3 (12.0)	I 5 (20.0)<br>II 16 (64.0)<br>III 4 (16.0)	0.917

Abbreviations: HTN, hypertension; DM, diabetes mellitus; HBV, hepatitis B virus; HCV, hepatitis C virus; EV, esophageal varices; AJCC, American Joint Committee on Cancer; BCLC, Barcelona Clinic Liver Cancer; PVT, portal vein thrombosis; MVI, macrovascular invasion; EHS, extrahepatic spread; MELD, Model for End-stage Liver Disease; FIB-4; fibrosis-4 index; ALBI, albumin-bilirubin; ERCP, endoscopic retrograde cholangiopancreatography; PTCD, percutaneous transhepatic cholangial drainage; RT, radiotherapy; TACE, transarterial chemoembolization; PSM, propensity score matching

**Data are presented as median (range). Categorical variables are presented as n (%). P values were calculated using Mann–Whitney U tests for continuous variables, Fisher's exact tests for binary categorical variables, and chi-square*

tests for multi-level categorical variables. Residual imbalance remained after matching for hypertension (absolute SMD = 0.783) and MELD score (absolute SMD = 0.731).

Supplementary Table S3. Individual systemic-treatment regimens, treatment lines, timing relative to biliary drainage, post-drainage treatment duration, and subsequent systemic therapies

**Patient**	**Systemic regimen**	**Systemic line**	**Timing relative to drainage**	**Post-drainage treatment duration, days**	**Subsequent systemic therapy**
1	Regorafenib	2	Before drainage	219	None
2	Lenvatinib	1	After drainage	88	Atezolizumab + bevacizumab
3	Sorafenib	1	After drainage	1143	None
4	Lenvatinib	1	After drainage	59	None
5	Sorafenib	1	After drainage	87	None
6	Sorafenib	1	After drainage	134	None
7	Pembrolizumab + bevacizumab	1	After drainage	63	Atezolizumab + bevacizumab

Patient	Systemic regimen	Systemic line	Timing relative to drainage	Post-drainage treatment duration, days	Subsequent systemic therapy
8	Nivolumab + bevacizumab	2	After drainage	72	None
9	Sorafenib	1	After drainage	84	None
10	Atezolizumab + bevacizumab	1	Before drainage	42	None
11	Atezolizumab + bevacizumab	1	Before drainage	68	Lenvatinib
12	Lenvatinib	1	After drainage	44	None
13	Sorafenib	1	After drainage	89	None
14	Atezolizumab + bevacizumab	1	After drainage	31	Pembrolizumab, followed by lenvatinib
15	Lenvatinib	2	After drainage	35	None
16	Atezolizumab + bevacizumab	1	After drainage	234	None
17	Nivolumab	2	After drainage	32	None
18	Regorafenib + pembrolizumab	2	After drainage	140	None
19	Pembrolizumab	3	After drainage	28	None
20	Atezolizumab + bevacizumab	2	Before drainage	33	None
21	Sorafenib	1	After drainage	269	None
22	Lenvatinib	1	After drainage	108	None
23	Lenvatinib	1	After drainage	110	Sorafenib
24	Sorafenib	1	Before drainage	106	None
25	Sorafenib	1	After drainage	70	Lenvatinib + pembrolizumab
26	Lenvatinib	1	After drainage	18	None
27	Nivolumab	1	After drainage	109	None
28	Nivolumab	2	After drainage	21	None
29	Sorafenib	1	After drainage	831	None
30	Sorafenib	1	After drainage	38	None

Supplementary Table S4. Sensitivity analyses of the association between systemic treatment and survival outcomes

Analysis	Outcome	Patients, n	Events, n	HR / adjusted HR	95% CI	*p*
Time-dependent Cox analysis	OS	78	62	0.70	0.40–1.22	0.207
	PFS	78	66	0.65	0.38–1.13	0.128
42-day landmark analysis	OS	63	50	1.07	0.57–2.04	0.826
	PFS	61	52	0.69	0.34–1.39	0.297

Analysis	Outcome	Patients, n	Events, n	HR / adjusted HR	95% CI	*p*
Cox model adjusted for radiotherapy	OS	78	62	0.55	0.32–0.93	0.027
	PFS	78	66	0.48	0.28–0.82	0.007
PSM cohort additionally adjusted for MELD score and hypertension	OS	50	37	0.68	0.34–1.35	0.265
	PFS	50	41	0.50	0.25–0.98	0.043

Abbreviations: *CI, confidence interval; HR, hazard ratio; MELD, Model for End-Stage Liver Disease; OS, overall survival; PFS, progression-free survival; PSM, propensity score matching. 62 deaths occurred and 16 patients were censored for OS; 66 progression/death events occurred and 12 patients were censored for PFS.*

Supplementary Table S5. Standardized mean differences before and after propensity-score matching

Variable	Type	SMD_before_PSM	SMD_after_PSM
Age	Contin.	-0.3838	-0.1029
Male Sex	Binary	-0.0667	0
Extrahepatic spread	Binary	0.15	0.12

Beyond up-to-11 criteria	Binary	0.1417	0
ALBI grade I	Binary	0.0958	0
ALBI grade II	Binary	0.2208	0.04
ALBI grade III	Binary	-0.3167	-0.04
Portal vein thrombosis	Binary	0.0667	0.04

Abbreviations: *SMD, standardized mean difference; PSM, propensity-score matching; ALBI, albumin–bilirubin. An absolute SMD <0.10 was used as the reference threshold for acceptable covariate balance.*
